# Mini open stent grafting with half sternotomy for aortic arch aneurysm

**DOI:** 10.1186/s13019-019-0923-x

**Published:** 2019-06-06

**Authors:** Tamaki Takano, Masayuki Sakaguchi, Takamitsu Terasaki, Taishi Fujii, Yusuke Date, Mugumi Fuke, Kai Machida

**Affiliations:** Department of Cardiovascular Surgery, Nagano Red-Cross Hospital, 5-22-1 Wakasato, Nagano, 3808582 Japan

**Keywords:** Open stent, Minimally invasive cardiac surgery, Half sternotomy

## Abstract

**Background:**

Open stent grafting is an alternative of graft replacement and thoracic endovascular aortic repair for aortic arch aneurysm. We have performed open stent grafting with half sternotomy (mini-OSG) to reduce in-hospital stay and recovery time of patients and herein report seven cases of mini-OSG for aortic aneurysm and dissection.

**Case presentation:**

The patients’ mean age was 66 years. Cardiopulmonary bypass was performed conventionally, and an open stent graft was inserted via an aortotomy on the aortic arch during circulatory arrest. No mortality occurred. The mean operation time was 387 min, and the mean blood loss was 587 ml. The patients were weaned from the ventilator 7.1 h postoperatively. No pseudoaneurysm or endoleakage was observed during the 2- to 20-month follow-up.

**Conclusions:**

Mini-OSG might be less invasive, although further studies and intensive follow-up are needed.

## Background

Total arch replacement (TAR) is still considered invasive. In one recent report of TAR, prolonged intubation was observed in 15.4% of patients and operative mortality was reported in 5.3% [1]. Open stent grafting (OSG) was introduced to obviate the need for distal anastomosis in the deep surgical field during TAR for aortic dissection [2]. Its clinical outcomes are reportedly acceptable, although no long-term analyses have been conducted [3]. One study showed that in patients undergoing aortic valve replacement, partial sternotomy reduced the length of stay in the intensive care unit and postoperative blood loss volume [4]. Since 2015, we have performed mini-OSG, which is a combination of OSG and partial sternotomy to reduce the operative risk by eliminating the distal anastomosis in aortic arch repair. We herein report the initial results of this procedure.

## Case presentation

**Fig. 1:**
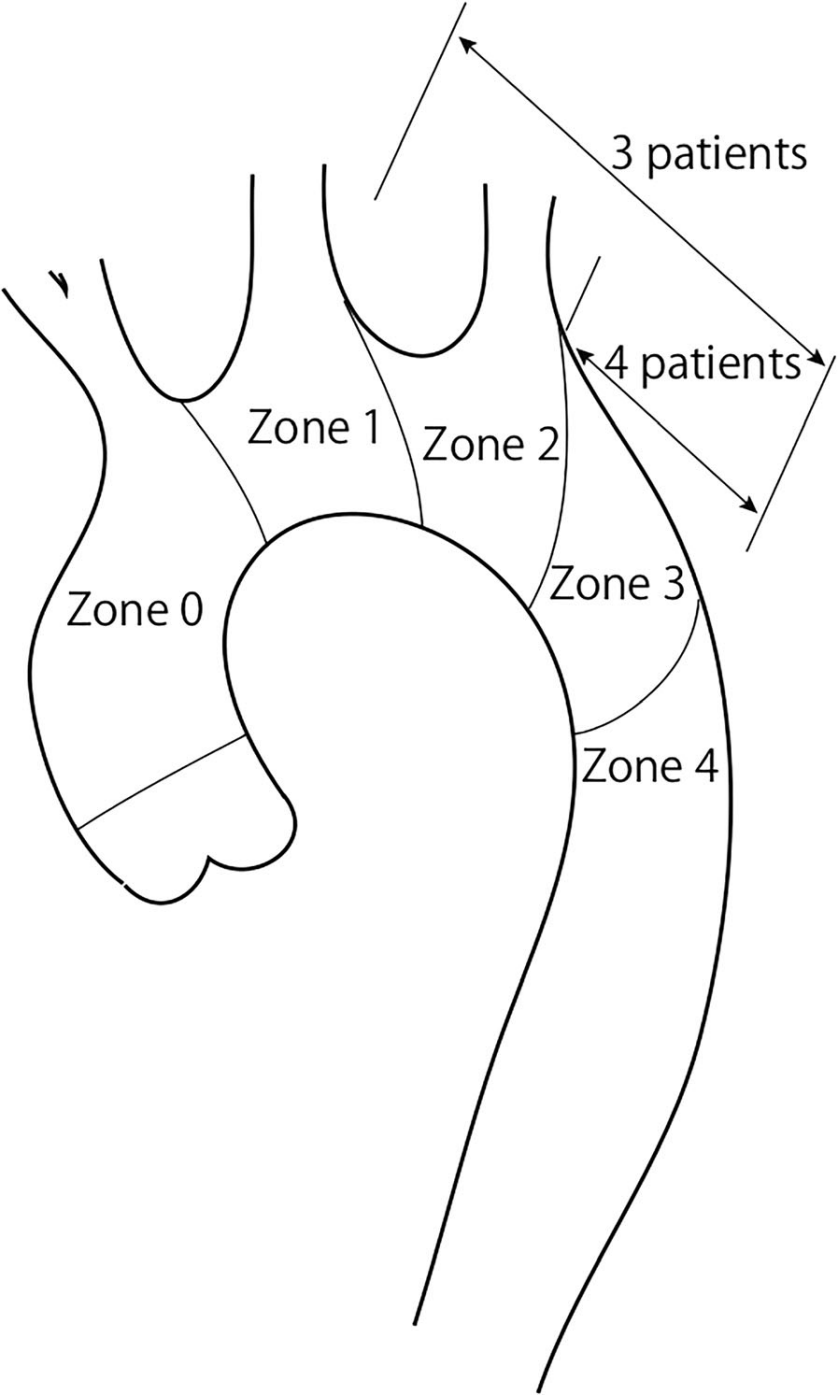
The aneurysm and entry point of the dissection were located in zone 3 in four patients and in zones 2 through 3 in three patients

Seven patients underwent mini-OSG in our institution beginning in March 2015. The aneurysm and entry point of the dissection were located in zone 3 in four patients and in zones 2 through 3 in three patients (Fig. 1) The patients’ mean age was 66 ± 19 years (range, 20–81 years), and all patients were male. The etiology was dissection in two patients and an aneurysm in five. Two patients underwent concomitant reconstruction of the left subclavian artery with a prosthetic graft, and one patient underwent left axillary artery reconstruction.

**Fig. 2:**
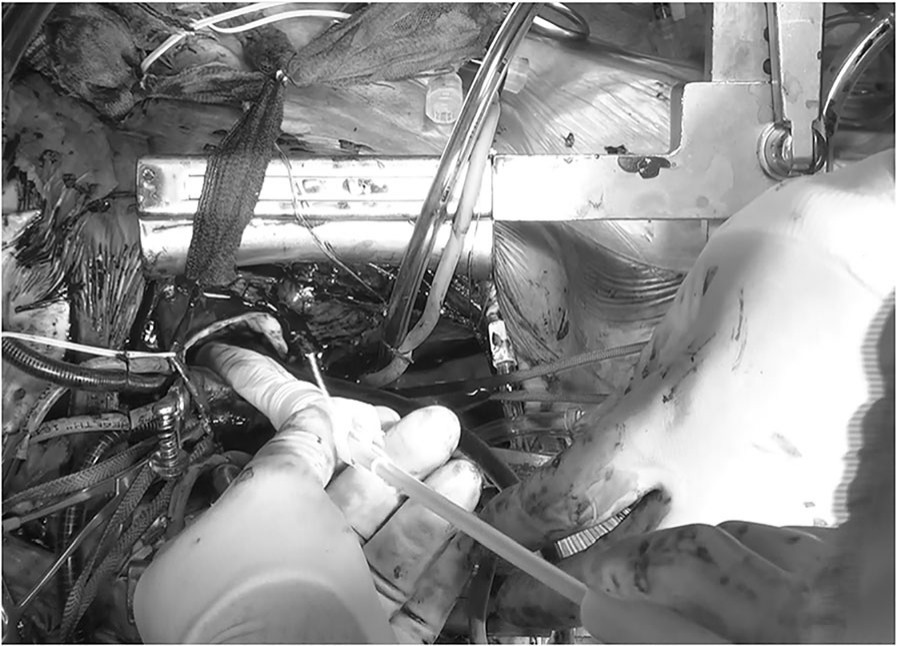
The open stent graft was inserted via the aortotomy during circulatory arrest

**Fig. 3:**
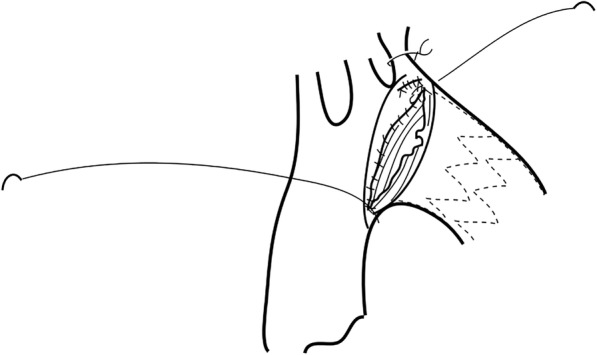
The proximal end of the stent was fixed on the aortic wall with a running suture. The left subclavian artery was ligated and reconstructed with a prosthetic graft

General anesthesia was induced in the same manner as in a standard sternotomy. An upper half sternotomy was performed until the fourth intercostal space was reached. Cardiopulmonary bypass was initiated with cannulation of the right axillary artery, ascending aorta, and right atrium. The femoral artery and vein were used when exposure was difficult because of the small surgical field. Left ventricular venting was performed via the left upper pulmonary vein or main pulmonary artery. After induction of moderate hypothermia at a rectal temperature of 24 °C to 28 °C, an aortotomy was performed on the anterior wall of the distal arch. Selective antegrade cerebral perfusion was then begun with the right axillary and left common carotid and subclavian arteries. An open stent graft (Japan Lifeline, Tokyo, Japan) measuring 21 to 33 mm in diameter and 6 or 9 cm in length was inserted through the aortotomy on the aortic arch during circulatory arrest (Fig. 2). The proximal end of the stent graft was directly sutured to the posterior wall of the aorta with a running suture, and the anterior wall of the stent was sutured during aortotomy closure (Fig. 3). When the aneurysm or dissection entry involved the left subcavian artery, a prosthetic graft with 9 mm diameter was anastomosed to the left subclavian artery by end to end fashion after the left subclavian artery was dissected. A prosthetic graft was anastomosed to the left axillary artery prior to the sternotomy when the aneurysm or dissection entry involved the left subcavian artery and there was not enough length of the subclavian artery for end to end anastomosis. The proximal end of the prosthetic graft was anastomosed to the ascending aorta after the open stent graft was inserted.

The mean operation time was 387 ± 70 min (range, 294–480 min). The mean cardiopulmonary bypass time and cardiac arrest time was 199 ± 48 min (range, 144–291 min) and 74 ± 33 min (range, 37–121 min), respectively. The mean selective cerebral perfusion time under circulatory arrest was 70 ± 29 min (range, 37–119 min). The mean intraoperative blood loss volume was 587 ± 226 ml. No patients required re-exploration for bleeding. The patients were weaned from the ventilator 7.1 ± 4.9 h (range, 2–11 h) postoperatively. The mean postoperative hospital stay and intensive care unit stay were 21 ± 7.4 (range, 13–33) and 3.2 ± 1.36 (range, 2–5) days, respectively. No mortality occurred. One patient developed left hemiplegia but recovered to normal activities of daily living. Follow-up computed tomography (CT) was performed 6 months after the surgery and then once a year. No pseudoaneurysm or endoleakage was observed during 2 to 20 months of follow-up.

## Discussion and conclusions

TAR with median sternotomy is a standard procedure for aortic arch surgery because cerebral perfusion is safely assured by antegrade cerebral perfusion and myocardial protection is easily performed. However, surgical exposure is sometimes limited and additional left thoracotomy might be required in cases aortic aneurysm or dissection entry exists beyond the left subclavian artery. In a recent report, extended TAR with the combination of left anterior thoracotomy and upper median sternotomy displayed a higher rate of respiratory complications compared to standard TAR with the median sternotomy [5]. Thoracic endovascular aortic repair is becoming applied for aortic arch pathologies. In-hospital mortality was reported as 4–5%, and cerebrovascular event occurred in 2–11% after the repair, which was better than the open surgery [6, 7]. However, 33% of patients underwent aorta-related reintervention during 9 months follow-up and estimated freedom from any aorta and/or non-aorta related reintervention at 12 months was 48 ± 13% [7]. OSG is considered advantageous compared to total arch replacement because the needs for a distal anastomotic suture and a left thoracotomy are eliminated. Additionally, in contrast to thoracic endovascular repair, stent detachment is minimized [8]. Historically, OSG was utilized to eliminate the distal anastomosis beyond the left subclavian artery during TAR for acute aortic dissection [2]. The aortic arch was dissected between the brachiocephalic and left common carotid arteries, and the graft was inserted in the dissected lumen toward the descending aorta, covering the carotid and subclavian arteries. The proximal end of the graft was sutured to the branched non-stented vascular graft, which replaced the ascending aorta and brachiocephalic artery with reconstruction of the brachiocephalic, left common carotid, and subclavian arteries. The frozen elephant trunk technique was introduced in 2001 by Karck et al. to facilitate thrombosis in the space between the graft and aorta, reducing the wall stress of the descending aorta [9]. The graft was delivered to the descending aorta, and the proximal site of the graft was directly sewn to the native aortic wall with or without ascending aorta and aortic arch replacement. We performed mini-OSG in the patients with aortic arch aneurysm and dissection, which involved the left common carotid and the subclavian arteries. In this initial series, no mortality was observed although one patient developed left hemiplegia. No pseudoaneurysm or endoleakage was observed during 2 to 20 months of follow-up. The mean intraoperative blood loss volume was 587 ml, and the patients were weaned from the ventilator 7.1 h postoperatively. These results suggest that mini-OSG might be an alternative treatment for aortic arch pathologies.

In our initial series of mini-OSG, we solely applied OSG via the aortic arch without graft replacement of the ascending aorta and aortic arch to simplify the procedures (Fig. 1). Preoperative CT revealed that the ascending aorta and aortic arch were not diseased in all patients, and the non-diseased ascending aorta and aortic arch were preserved during the surgery. Replacement of the non-diseased aorta in patients with distal arch lesions remains controversial. In our institution, distal arch replacement through a median sternotomy had been performed with preservation of the healthy proximal arch and neck vessels for treatment of distal arch aneurysm. We reported the early operative results and late outcomes of this procedure, including changes in the size of the remaining arch and thoracic aorta [10]. Twenty-three patients underwent elective distal arch replacement through a median sternotomy. The proximal end of the aneurysm was located in zone 1 in 21.7% of the patients, zone 2 in 39.1%, and zone 3 in 39.1%. With selective cerebral perfusion and hypothermic circulatory arrest, distal arch was replaced with prosthetic graft without reconstruction of the ascending aorta. Neither operative mortality nor postoperative low cardiac output syndrome occurred in all 23 patients. Two patients (8.7%) developed perioperative stroke. In the late period, two cardiovascular-related deaths occurred; one was due to rupture of the aneurysm in the descending aorta, and the other was due to heart failure. Postoperative CT showed that the maximal aortic diameter of the proximal arch increased by 0.8 ± 1.9 mm while that of the distal arch increased by 3.9 ± 9.4 mm during a postoperative follow-up period of 9.2 ± 4.7 years. These results suggest that the preserved aortic arch in mini-OSG might not influence adverse outcomes, although the number of patients was small in our previous study and careful observation is required after the surgery.

In conclusion, we performed mini-OSG with upper-half sternotomy in seven patients. This procedure might be less invasive, although further studies with large numbers of patients and intensive follow-up are needed.

## Data Availability

The datasets and materials in this report are available from the corresponding author on appropriate requests.

## Abbreviations

Mini-OSG: Open stent grafting with half sternotomy
OSG: Open stent grafting
TAR: Total arch replacement
